# A retroperitoneal abscess caused by *Haemophilus parainfluenza *after endoscopic retrograde cholangiopancreatography and open cholecystectomy with a common bile duct exploration: a case report

**DOI:** 10.1186/1752-1947-4-170

**Published:** 2010-06-03

**Authors:** Shonak B Patel, Zubair A Hashmi, Robert J Marx

**Affiliations:** 1 Northside Medical Center, Department of Surgery, 500 Gypsy Lane, Youngstown, OH 44505, USA

## Abstract

**Introduction:**

Abscesses after open cholecystectomies have been reported to occur in less than 1% of patients. The majority of these abscesses are colonized by gastrointestinal tract flora. It is clearly known that *Haemophilus parainfluenza *is a normal inhabitant of the human respiratory tract. However, its origin and route of transmission into the gastrointestinal tract is unknown.

**Case presentation:**

We present the case of a 68-year-old Caucasian female who developed a retroperitoneal abscess caused by *H. parainfluenza *after open cholecystectomy and common bile duct exploration. This presented nearly five weeks post-operatively. She underwent a second operation to drain the abscess, and was subsequently placed on appropriate antibiotics.

**Conclusion:**

A retroperitoneal abscess due to *H. parainfluenza *is extremely rare. It is a normal inhabitant of the human respiratory tract. To the best of our knowledge, there have been only a few reported cases of these abscesses, and they mainly involve the psoas muscle. The retroperitoneal abscess originated from the oropharynx, most likely after the endoscopic retrograde cholangiopancreatography was performed. With the advent of Natural Orifice Translumenal Endoscopic Surgery, oral decontamination will need to be considered to decrease the potential for such infections.

## Introduction

*Haemophilus parainfluenza *is generally regarded as a commensal bacterium in the respiratory tract. It has been known to provoke respiratory tract infections, otitis, and meningitis. However, little is known about its ability to colonize other sites. A search of the literature reveals only one reported case of *H. parainfluenza *in the gastrointestinal tract [1]. We now report a second case in which a 68-year-old presented with a retroperitoneal abscess due to *H. parainfluenza *after open cholecystectomy and common bile duct (CBD) exploration.

### Case presentation

A 68-year-old Caucasian female with a history of hypertension and hysterectomy (approximately 20 years ago) presented to the hospital with dehydration and right upper quadrant pain. A computed tomography (CT) scan of our patient obtained as an out-patient showed irregular thickening of the gallbladder wall associated with stones, but there was no evidence of cholecystitis. Both the intrahepatic and extrahepatic biliary ducts were dilated approximately to the level of the CBD, but indicated no choledocolithiasis. A follow-up endoscopic retrograde cholangiopancreatography (ERCP), performed to evaluate for possible cholangiocarcinoma, revealed a CBD stone with marked intrahepatic and extrahepatic duct dilatation. ERCP indicated no evidence of a cholangiocarcinoma. After papillotomy, the endoscopist determined the stone was beyond the scope of endoscopic removal, thus open cholecystectomy with CBD exploration was indicated.

Two days later, our patient underwent an open cholecystectomy and CBD choledochoscopy with an Olympus VRF Type P2™ flexible fiberoptic choledocoscope. A prophylactic antibiotic, 1 g cefazolin, was given on induction of anesthesia. The choledocolith was removed by choledocotomy performed on the palpable stone. A CBD T-tube was placed, brought out through the lateral abdominal wall, and attached to the bile drainage bag. No other stones remained in the CBD after the completion of surgery, which was confirmed by intra-operative cholangiogram. The stone was assumed to have been present in the CBD for many years since it was significantly large. The possibility of it being a primary CBD stone or a stone that was passed into the CBD years ago and continued to grow also had to be entertained.

**Figure 1:**
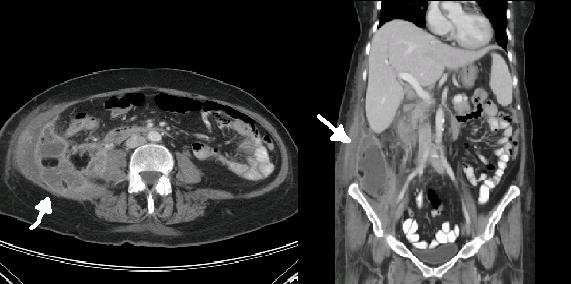
**White arrows indicating retroperitoneal abscess**.

Five weeks post-operatively, our patient was readmitted with dehydration and poor oral intake. The patient had remained afebrile with white blood cell count of 15.1×10^3^k/mcL with no shift in white blood cell morphology. A CT-scan was obtained and indicated a fluid collection suggesting an abscess of the right flank (figure 1). Our patient was taken to the operating room where a retroperitoneal abscess was found that tracked into the right paracolic gutter and into the right flank. There was no subhepatic fluid collection. The CBD repair was intact. Multiple drains were placed in the abscess cavity. Cultures of the collected fluid revealed *H. parainfluenza*. An Infectious Disease specialist was consulted, and the patient was placed on intravenous ceftriaxone (which was susceptible to the organism) for 6 weeks. Follow-up appointments and CT-scans at 6 weeks showed a decrease in the fluid collection, and she denied any further symptoms.

## Discussion

A retroperitoneal abscess due to *H. parainfluenza *is very rare. There have been only a few reported cases of these abscesses, mainly involving the psoas muscle [2,3]. One case reports a retroperitoneal abscess caused by *H. parainfluenza *after ERCP [1]. According to Riahi *et al*., possible explanations for the abscess include infection secondary to retroperitoneal perforation occurring during sphincterotomy or introduction of the bacterium from the upper airway during the ERCP and subsequently to the retroperitoneal space via a perforation in the performance of the sphincterotomy [1]. Our case also involved ERCP with papillotomy, a possible port of entry for the bacterium. The common duct stone was too large (3 to 4 cm) to remove via ERCP; therefore an open cholecystectomy and CBD exploration was performed. If *H. parainfluenza *was introduced during the ERCP, there was no evidence of perforation or abscess and the CBD was intact at operation. It is well understood that *H. parainfluenza *is a common inhabitant of the mucosal surfaces of the human upper respiratory tract [4]. There is a possibility that the patient may have subsequently coughed up and swallowed the *H. parainfluenza*, moving it into the gastrointestinal tract and allowing it to track back along the T-tube. Another possibility may be that *H. parainfluenza*, is a rarely identified bacterium found in the intestinal tract of healthy patients, and seldom causes problems until surgical manipulation allows it to manifest as an abscess. Our patient had no other source of infection (upper respiratory, pneumonia, otitis, etc), nor did the abscess grow any other organism other than *H. parainfluenza.*

## Conclusions

Abscesses after open cholecystectomies have been reported to occur in less than 1% of patients [5]. The majority of these abscesses are colonized by gastrointestinal tract flora [5]. *H. parainfluenza *is a normal inhabitant of the human respiratory tract and its route of translocation into the gastrointestinal tract is largely unknown. We postulate that, in this case, the *H. parainfluenza *found in the retroperitoneal abscess originated from the oropharynx and may have been introduced from the ERCP. We conclude that investigations for *H. parainfluenza *should be performed more often in relation to the intestinal tract. With the advent of NOTES, or Natural Orifice Translumenal Endoscopic Surgery, oral decontamination [5] may need to be considered to decrease the potential for such infections.

### Consent

Written informed consent was obtained from the patient for publication of this case report and any accompanying images. A copy of the written consent is available for review by the Editor-in-Chief of this journal.

## Competing interests

We have nothing to declare or disclose. No competing interest to declare.

## Authors' contributions

SP carried out the search and obtained data on *H. parainfluenza*, and cross referenced its occurrence to extra-respiratory infections, assisted in compiling the manuscript, and also carried out chart review, as well as obtaining images. ZH carried out the search and obtained data on *H. parainfluenza*, and cross referenced its occurrence to extra-respiratory infections, assisted in compiling the manuscript, and also carried out chart review, as well as obtaining images. RM provided the editing, along with assisting in compiling the manuscript. SP, ZH, RM were all directly involved in both operations, as well as the post-operative care and management of the patient. All authors have read and approved the final manuscript.
